# 

**Published:** 1793

**Authors:** 


### CONTENTS.

Page

I. *OBSERVATIONS on the Fevers and Dyſentery of hot Climates; and on the Uſe of Mercury in thoſe Diſeaſes. By Mr.* William Boag, *Surgeon in the Service of the Honourable Eaſt-India Company at Bombay.* — — — 1

II. *An Account of the ſucceſsful Treatment of a Caſe in which the Brachial Artery was divided. By* William Adair, *Eſq. Surgeon General to the Garriſon of Gibraltar.* — — — — 21

III. *An Account of the Effects of Oil of Turpentine in a Caſe of internal Hæmorrhage. By the ſame.* — — 25

IV. *A Caſe of Imperforated Anus. By the ſame.* — — — — 27

V. *Obſervations on the Pathology and Mode of Treatment of Calculi in general, but*

more

Page

*more particularly of Inteſtinal Calculi; with a Deſcription and Chemical Analyſis of the Inteſtinal Calculi of Horſes. By Mr.* William Gaitſkell, *Surgeon at Rotherhithe.* — — — 31

VI. *An Account of the good Effects of Opium in a Caſe of Retention of Urine. By Mr.* Alexander Mather, *Surgeon at York.* 102

VII. *A Caſe of monſtrous Birth. By the ſame.* — — — 107

VIII. *A Caſe of Varicoſe Aneuriſm. By Mr.* H. Park, *Surgeon to the Liverpool Infirmary.* — — — — 111

IX. *An Account of the good Effects of Opium adminiſtered in Clyſters, in Caſes of Menorrhagia. By Mr.* Peter Copland, *Surgeon at Swayfield, near Colſterworth, in Lincolnſhire.* — — — 118

X. *An Account of the good Effects of a Mercurial Snuff in a Caſe of Gutta Serena. By Mr.* R. B. Blagden, *Surgeon at Petworth, in Suſſex.* — — 126

Page

XI. *A Case of Pulmonary Hæmorrhage, with Remarks. By Mr.* William Davidson, *Apothecary in London.* — — 129

XII. *A Case of Psoas Abscess successfully treated. By Mr.* William Smith, *Surgeon at Bideford, and Member of the Corporation of Surgeons of London.* — 138

XIII. *Case of Phlegmonic Inflammation, with Reflections on certain Effects of Heat and Cold on the living System. By* Thomas Beddoes, *M. D.* — — — 148

XIV. *Observations on the good Effects of Caustics in Cases of White Swellings of the Joints. By Mr.* Bryan Crowther, *Surgeon to Bridewell and Bethlem Hospitals.* 157

XV. *On the Cure of the Elephantiasis. By* At'har Ali' Khán, *of Dehli. From the Asiatick Researches: or, Transactions of the Society instituted in Bengal, for inquiring into the History and Antiquities, the Arts, Sciences, and Literature of Asia.* — 168

XVI. *On the Spikenard of the Ancients. By Sir* William Jones, *Knt. From the same Work.* — — — 180

Page

XVII. *An Account of ſome chemical Experiments on Tabaſheer. By* James Louis Macie, *Eſq. F. R. S. From the Philoſophical Tranſactions of the Royal Society of London.* — — 193

*Catalogue of Books.* — — 200

*Index.* — — 225

